# Association of Gene Variants with Seasonal Variation in Muscle Strength and Aerobic Capacity in Elite Skiers

**DOI:** 10.3390/genes14061165

**Published:** 2023-05-26

**Authors:** Benedikt Gasser, Walter O. Frey, Paola Valdivieso, Johannes Scherr, Jörg Spörri, Martin Flück

**Affiliations:** 1 Department of Sport, Physical Activity and Health, University of Basel, 4052 Basel, Switzerland; 2 Hirslandenklinik, 8032 Zurich, Switzerland; walter.frey@hin.ch; 3 Laboratory for Muscle Plasticity, Balgrist Campus, University of Zurich, 8008 Zurich, Switzerland; valdy75@yahoo.it; 4 University Centre for Prevention and Sports Medicine, Department of Orthopaedics, Balgrist University Hospital, University of Zurich, 8008 Zurich, Switzerland; johannes.scherr@balgrist.ch (J.S.); joerg.spoerri@balgrist.ch (J.S.); 5 Sports Medical Research Group, Department of Orthopaedics, Balgrist University Hospital, University of Zurich, 8008 Zurich, Switzerland; 6 Department of Medicine, University of Fribourg, 1700 Fribourg, Switzerland; 7 Swiss Federal Institute of Sport—BASPO, 2532 Magglingen, Switzerland

**Keywords:** periodization, eccentric and concentric muscle metabolism, age, sex

## Abstract

*Background:* The training of elite skiers follows a systematic seasonal periodization with a preparation period, when anaerobic muscle strength, aerobic capacity, and cardio-metabolic recovery are specifically conditioned to provide extra capacity for developing ski-specific physical fitness in the subsequent competition period. We hypothesized that periodization-induced alterations in muscle and metabolic performance demonstrate important variability, which in part is explained by gene-associated factors in association with sex and age. *Methods:* A total of 34 elite skiers (20.4 ± 3.1 years, 19 women, 15 men) underwent exhaustive cardiopulmonary exercise and isokinetic strength testing before and after the preparation and subsequent competition periods of the World Cup skiing seasons 2015–2018. Biometric data were recorded, and frequent polymorphisms in five fitness genes, ACE-I/D (rs1799752), TNC (rs2104772), ACTN3 (rs1815739), and PTK2 (rs7460, rs7843014), were determined with specific PCR reactions on collected DNA. Relative percentage changes of cardio-pulmonary and skeletal muscle metabolism and performance over the two seasonal periods were calculated for 160 data points and subjected to analysis of variance (ANOVA) to identify hypothesized and novel associations between performance alterations and the five respective genotypes and determine the influence of age × sex. A threshold of 0.1 for the effect size (h2) was deemed appropriate to identify relevant associations and motivate a post hoc test to localize effects. *Results:* The preparation and competition periods produced antidromic functional changes, the extent of which varied with increasing importance for anaerobic strength, aerobic performance, cardio-metabolic efficiency, and cardio-metabolic/muscle recovery. Only peak RER (−14%), but not anaerobic strength and peak aerobic performance, and parameters characterizing cardio-metabolic efficiency, differed between the first and last studied skiing seasons because improvements over the preparation period were mostly lost over the competition period. A number of functional parameters demonstrated associations of variability in periodic changes with a given genotype, and this was considerably influenced by athlete “age”, but not “sex”. This concerned age-dependent associations between periodic changes in muscle-related parameters, such as anaerobic strength for low and high angular velocities of extension and flexion and blood lactate concentration, with rs1799752 and rs2104772, whose gene products relate to sarcopenia. By contrast, the variance in period-dependent changes in body mass and peak VO2 with rs1799752 and rs2104772, respectively, was independent of age. Likely, the variance in periodic changes in the reliance of aerobic performance on lactate, oxygen uptake, and heart rate was associated with rs1815739 independent of age. These associations manifested at the post hoc level in genotype-associated differences in critical performance parameters. ACTN3 T-allele carriers demonstrated, compared to non-carriers, largely different periodic changes in the muscle-associated parameters of aerobic metabolism during exhaustive exercise, including blood lactate and respiration exchange ratio. The homozygous T-allele carriers of rs2104772 demonstrated the largest changes in extension strength at low angular velocity during the preparation period. *Conclusions:* Physiological characteristics of performance in skiing athletes undergo training period-dependent seasonal alterations the extent of which is largest for muscle metabolism-related parameters. Genotype associations for the variability in changes of aerobic metabolism-associated power output during exhaustive exercise and anaerobic peak power over the preparation and competition period motivate personalized training regimes. This may help to predict and maximize the benefit of physical conditioning of elite skiers based on chronological characteristics and the polymorphisms of the ACTN3, ACE, and TNC genes investigated here.

## 1. Introduction

The yearly season periodization of skiers follows a specific pattern with the aim of peaking skiing-specific performance within the autumn to winter months of the competition period. This is facilitated by building up the required physical capacity in the preparation period during the spring and summer months [1,2] before the competition period begins in mid-November and continues into mid-April [1,2]. Throughout these periods, there is a variable contribution between on-snow and off-snow training, the latter of which is the main focus of the months from May to July before being mixed between August and October [1,2].

Physiologically, both Alpine and Nordic skiing place high demands on different organ systems, from the musculoskeletal to the cardiopulmonary systems [1,3,4]. Therefore, high values of “anaerobic” leg muscle strength during both extension and flexion and aerobic power, in addition to coordination skills, are favorable physiological qualities determining elite skiing performance [5,6,7,8,9,10,11,12,13,14,15,16]. These physiological characteristics can be considered a result of gene-environment interactions that affect adaptation to rigorous multiyear, year-round training programs [1], especially as genetic factors affect the anatomic design and training-related plasticity of functional structures [1,5,6,7,8,9,17,18].

Few studies have reported on the physiological characteristics of athletes over an entire skiing season. The available data emphasize that the physiological performance of elite Nordic and Alpine skiers demonstrates distinct intra-seasonal variability for aerobic parameters that may set the efficiency of energy use during intense exercise [19,20]. The extent to which observed antidromic adjustments of muscle performance and metabolism over the preparation and competition period and through years of training vary in association with genetic factors is not fully understood.

Combinations of gene variants plausibly affect skill levels in athletes beyond what is known for the “general” population and are differentially expressed over the season [10,17,21,22,23,24,25,26,27]. In this respect, frequent natural polymorphisms in genes affect the specialization of the metabolic and contractile phenotypes in healthy subjects and athletes with endurance- and strength-type training [10]. Namely, this concerns gene polymorphisms of angiotensin converting enzyme (ACE, insertion/deletion, rs1799752), tenascin-C (TNC, rs2104772, (A/T), actinin-3 (ACTN3, rs1815739, C/T), and protein tyrosine kinase 2 (PTK2, rs7460, rs7843014) that affect the fibre type composition, cross-sectional area, specific force, and capillary and mitochondria-related energy supply of the skeletal muscle and the myocard contractile performance [10,26,28,29,30,31]. Recently, the association of cardiopulmonary and muscle performance in elite skiers was reported with the aforementioned genotypes [26], but whether these differences are explained by exercise conditioning or progression through years of training has not yet been explored. The chosen genotypes were based on research on molecular and cellular mechanisms underpinning the associations of these genotypes with variability in performance in human investigations and transgenic experiments [32,33,34]. In consequence, in the current study, we investigated the extent to which intra-seasonal changes in peak values for muscle strength and indices of cardiovascular performance, especially those associated with the contribution of aerobic metabolism to power output, are associated with sequence variants in the ACTN3, PTK2, ACE-I/D, and TNC genes in elite skiers. Care was taken to assess the possible influence of the given factors, chronological age and sex, on identifiable associations between performance alterations over the preparation and competition periods and the five investigated genotypes. Our hypothesis for falsification was that (i) there is no difference in performance capacity before versus after a respective seasonal period and (ii) there is no interaction effect with the genotype on aspects of skeletal muscle and cardiovascular performance parameters for such periodic changes [35] (see Supplemental File S1). Therefore, specifically focusing on the alterations over the preparation and competition period for the relationships between (aerobic) metabolic parameters and power output during exhaustive exercise is important, as such parameters have been found to contribute to seasonal adaptations in skiing athletes [20].

## 2. Material and Methods

*Study Design***:** The investigation followed a cross-sectional design, whereby elite skiers were recruited during routine medical appointments. Biological data were gathered in routine test sessions before and after the preseason preparation and competition season periods (i.e., the preparation and competition periods) of the skiing seasons in 2015–2018, respectively. The study was conducted in line with the Declaration of Helsinki and approved by the local Ethics Committee of the Canton of Zurich (Switzerland)—BASEC Number: 2018-01598.

*Subjects*: Forty-three members of the Alpine (skiers from all Alpine subdisciplines) and Nordic (biathletes or cross-country skiers) skiing squads of a national team of a world-leading skiing nation participated in the study. The majority of subjects originated from alpine areas and followed the generic training scheme and recommendations for the type, volume, and intensity of exercise and competition over the different periods of the skiing season. This comprised a large degree of isometric strength exercise during the preparation period, varying degrees of training in different intensity zones (HIIT/circuit and aerobic training), and increasing amounts of eccentric contractions with skiing [1].

*Isokinetic strength tests*: Anaerobic peak performance of single contractions was measured with a CON-TREX^®^ isokinetic dynamometer (multijoint module 2100 CON-TREX-MJ with power module 1100 CON-TREX-PM-1, CMV AG, Dübendorf, Switzerland). The maximal power and moments of single contractions were measured during isokinetic leg extension and flexion at two angular velocities (60° s^−1^ and 360° s^−1^). The device was calibrated per the manufacturer’s specifications and verified prior to testing.

*Cardiopulmonary exercise*: Exhaustive exercise was performed with a ramp protocol on a cycling ergometer (for Alpine skiers) or treadmill (for Nordic skiers) under standardized conditions according to a published protocol by the Swiss Olympic Committee [36]. In brief, the test for Alpine skiers started with at least 2 min of rest when subjects sat still on the cycle ergometer while subsequently maintaining a normal breathing pattern without warming up, as this would have affected ACE activity [37]. Subjects started exercising at an initial power before the target power was increased in increments until subjects experienced volitional exhaustion and/or were not able to maintain the target pedal cadence and power output. Subsequently, recordings continued for a period of eight minutes when subjects rested in a seated position. Cardiopulmonary parameters were continuously monitored with a Metalyzer 3B-R2 device (Cortex, Leipzig, Germany), including measurements of cardiopulmonary parameters. The rate of perceived exertion was assessed each min with the Borg scale [25].

*Genotyping*: Gene polymorphisms of interest (ACE-I/D, rs1799752; ACTN3, rs1815739; TNC, rs2104772; PTK2, rs7460, and rs7843014) were determined from drawn blood with polymerase chain reactions (PCR) essentially as described [26]. In brief, blood was drawn from the cubital vein, and genomic DNA was extracted by the DNeasy Blood and Tissue Kit (Cat No. 69504, Qiagen, Basel, Switzerland). DNA concentration and purity were quantified using a NanoDrop USV-99 AGTGene (Labgene Scientific, Châtel-St-Denis, Switzerland), and DNA samples were diluted to a final concentration of 5 ng/μL for storage at −20 °C. PCRs were carried out with 10 ng genomic DNA and specific combinations of primers (0.2 μM), 2.5 mmol MgCl2, and KAPA HRM FAST Master Mix (KAPA BIOSYSTEMS, Labgene Scientific, Châtel-St-Denis, Switzerland) on a real-time PCR system (EcoTM, Illumina, San Diego, CA, USA, distributed by Labgene Scientific, Châtel-St-Denis, Switzerland). The generic reaction conditions were as follows: 3 min enzyme activation at 95 °C, followed by 35 amplification cycles (5 s denaturation at 95 °C and 30 s annealing/extension at 60 °C), and a final melting cycle (initiated by heating to 95 °C and a thermal ramp after cooling to 55 °C to 95 °C). Specific genotypes were determined based on high-resolution melting curve analysis with genetic variation analysis software (EcoStudy Version 5.0, Illumina R, San Diego, CA, USA). Negative, non-template control reactions, and positive controls comprising genotyped DNA samples for each variant were run in parallel PCRs. Where indicated, the genotype was confirmed by Sanger sequencing (Microsynth, Balgach, Switzerland) and gel electrophoresis. The following primer pairs were used as described [26,27]: rs1799752 (I-allele: 5′-tgggattacaggcgtgatacag-3′ and 5′ atttcagagctggaataaaatt-3′; D-allele: 5′-catcctttctcccatttctc-3′ and 5′-atttcagagctggaataaaatt-3′), rs2104772 (5′-caaaaagcagtctgagccac-3′ and 5′-ttcagtagcctctctctgagac-3′), rs1815739 (5′-ctgtttgcctgtgtgtaagtggggggg-3, 5′-tgtcacagtatgcaggagggg-3′), rs7460 (5′-tgggtcgggaactagctgta-3′, 5′-atggaaaaaggggatggtcc-3′), and rs7843014 (5′-tgatgggacctaaacccatt-3′, 5′-tttcccatcagctgcttgtt-3′). For further details, see Supplemental Table S1 and Supplemental Figure S4 [26,27].

*Data handling*: The results for parameters of anaerobic performance from isokinetic strength testing (see abbreviation list) and aerobic performance from cardiopulmonary exercise testing (see abbreviation list) were imported into MS-Excel (Microsoft Office Professional Plus 2019, Microsoft Inc., Redmond, WA, USA) and anonymized. Data were assessed for the presence of missing or inconsistent values, and the slopes of metabolic parameters versus produced power during cardiopulmonary exercise were calculated as described [36]. As missing values were below five percent in almost all cases, no imputing was performed [36]. The area(s) under the curves (AUC) for the produced work (Watt × s) and accumulating lactate (mM × s) were estimated using geometry formulas for rectangles and triangles for the incremental areas of the respective sampling interval. Subsequently, data sets that covered consecutive seasonal periods were identified, and periodic changes over the preparation period and the competition period were calculated (from the percentage difference in values between measurements in autumn and spring of the same year and between measurements in spring and autumn from the preceding year, respectively). Data were missing in instanced for certain athletes due to injury, entry in, or exit from the squat. The repetitive testing over the four skiing sessions yielded eighty and one hundred twenty-five measurements, respectively, from thirty-four elite skiers (fifteen males and nineteen females), which were subjected to statistical analysis. A total of 166 and 39 data points were from alpine and Nordic skiers, respectively.

*Statistics*: The statistical significance of changes over a seasonal period was assessed with a sign test. Descriptive statistics shown in figures are illustrated with quartiles. Factorial influences on periodic changes were tested with univariate analysis of variance for the factors period (preparation period, competition period) × genotype (rs1799752, rs1815739, rs2104772, rs7460, and/or rs7843014) × age × sex. For effect localization, a test for the least significant difference was used. Interaction effects of the genotype were assessed by carrying out multiple ANOVAs for the combinations (rs1799752 × rs1815739, rs1799752 × rs2104772, and rs1815739 × rs7460 × rs7843014) because certain genotype combinations were too rare to completely compute the model. Relevance was determined by the presence of an effect size (h2 > 0.1) and the statistical significance of the influence *(p* < 0.05). Analyses were performed in SPSS (version 28.0.0.0, IBM).

## 3. Results

***Alterations in performance over the preparation and competition periods of an entire skiing season*:** Table 1 summarizes the physiological data for the first skiing season of the thirty-four elite skiers for which data from at least one consecutive cycle of one preparation period and one competition period was available for the skiing seasons in 2015–2018.

Only peak RER (−14%) and age, but not anaerobic strength and peak aerobic performance, before the preparation period differed between the first and the last year. Conversely, slopes of the linear relationship between power output and metabolic parameters during cardiopulmonary exercise to exhaustion, which characterize cardio-metabolic efficiency, were not different between the preparation period in the first and last year of the investigated skiing seasons, i.e., slopes of P vs. VO2 (*p* = 0.120), vs. HR (*p* = 0.405), vs. VE (*p* = 0.068), and vs. RER (*p* = 0.054).

**Figure 1:**
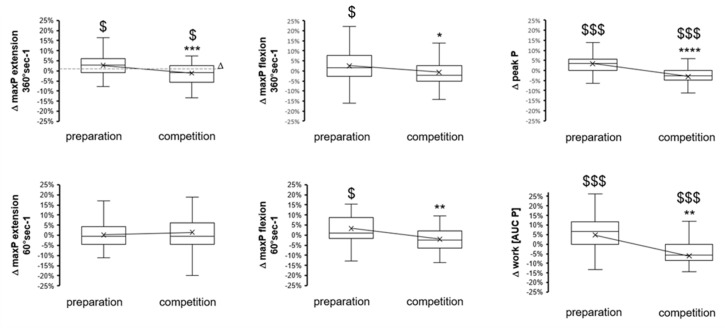
Box-whisker plots of the changes in performance over the preparatory and competition periods of the yearly skiing season. $, $$$, *p* < 0.05, 0.005, 0.001, for the effect of the seasonal period (sign test). *, **, ***, **** *p* < 0.05, 0.01, 0.005, 0.001 vs. the preparation period, respectively. ANOVA with a post hoc test of least significance.

**Figure 2:**
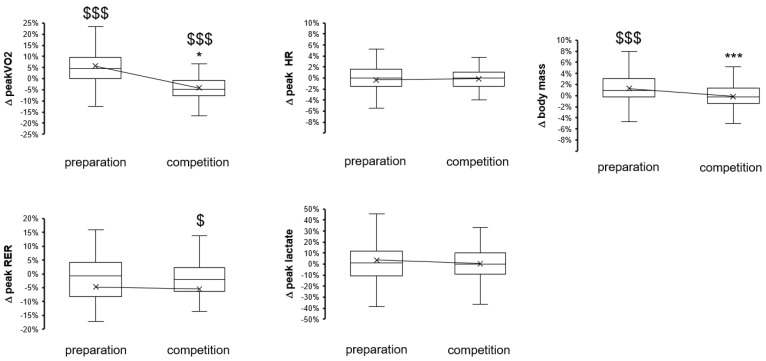
Box-whisker plots of the changes in peak values for metabolic parameters during exhaustive ramp exercise over the preparatory and competition periods of the yearly skiing season. $, $$$, *p* < 0.05, 0.001, for the effect of the seasonal period (sign test). *, ***, *p* < 0.05, 0.005, vs. the preparation period, respectively. ANOVA with a post hoc test of least significance.

**Figure 3:**
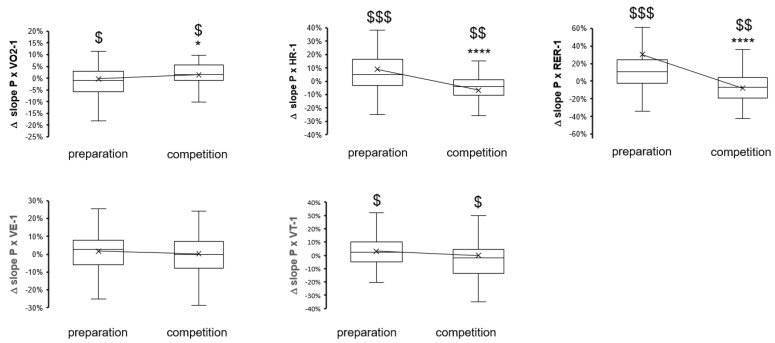
Box-whisker plots of the changes in slopes of produced power per metabolic parameter during exhaustive ramp exercise over the preparatory and competition periods of the yearly skiing season. $, $$, $$$, *p* < 0.05, 0.01, 0.005, 0.001, for the effect of the seasonal period (sign test). *, **** *p* < 0.05, 0.001 vs. the preparation period, respectively. ANOVA with a post hoc test of least significance.

**Figure 4:**
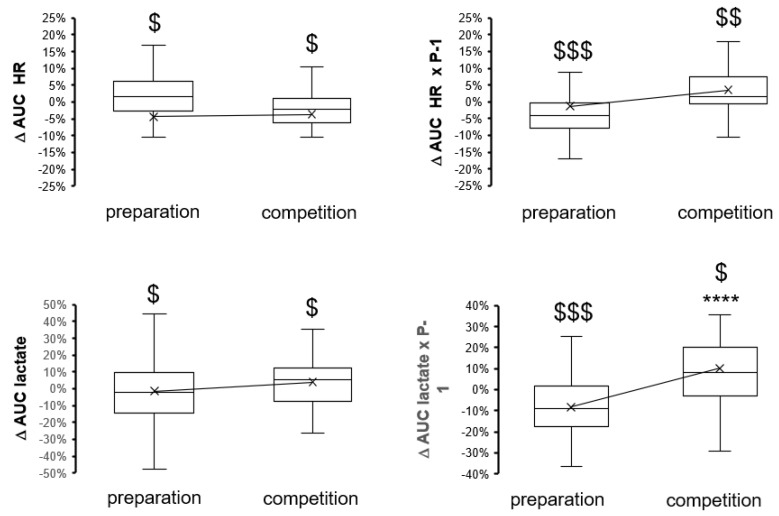
Box-whisker plots of the changes in the AUC of metabolic parameters (and, power-related values) during exhaustive ramp exercise over the preparatory and competition periods of the yearly skiing season. $, $$, $$$, *p* < 0.05, 0.01, 0.005, 0.001, for the effect of the seasonal period (sign test). *, **, ***, **** *p* < 0.05, 0.01, 0.005, 0.001 vs. the preparation period, respectively. ANOVA with a post hoc test of least significance.

In general, the preparation and competition periods were associated with antidromic functional adaptations. Muscle performance and underpinning metabolic function improved over the preparation period and were deconditioned over the subsequent competition season period (Figure 1, Figure 2, Figure 3 and Figure 4). Indices of anaerobic power, i.e., max P in leg extension and flexion at 360° s^−1^, were improved by 3% during the preparation period but deteriorated over the subsequent competition period (Figure 1). Peak aerobic power improved by 6% during the preparation period and likewise deteriorated during the subsequent competition period. Body mass increased by two percent over the preparation period compared to an overall unchanged body mass over the competition period (Figure 2).

Correspondingly, metabolic aspects also changed over the two seasonal periods. Peak values of maximal oxygen uptake (Δ peak VO2) increased over the preparation period and deteriorated over the subsequent competition season period (Figure 2). Peak values for heart rate and RER decreased over the preseason preparation period, but this change was indifferent to the change over the competition season period.

Interseasonal variability was also noted for parameters that reflect the reliance of power output on cardiopulmonary and muscle parameters: The slope of power produced per VO2 and the power-related values for blood lactate concentration both decreased over the preseason preparation period (Figure 3 and Figure 4), and the power-related values for blood lactate concentration were thereafter increased over the competition season period. Conversely, the slopes of power output per heart rate and per RER were elevated over the preparation period and reduced over the competition period. Peak values of heart rate and lactate, respectively, and their integrals were little and indifferently affected between the preparation and competition periods (Figure 2 and Figure 4).

Metabolic recovery after exhaustive exercise was also affected by the seasonal period. Two minutes after ramp exercise, heart rate and blood lactate concentration demonstrated lowered values after the preseason preparation period. However, the recovery to lower values attenuated the heart rate after the competition season period (Figure S1).

**Figure 5:**
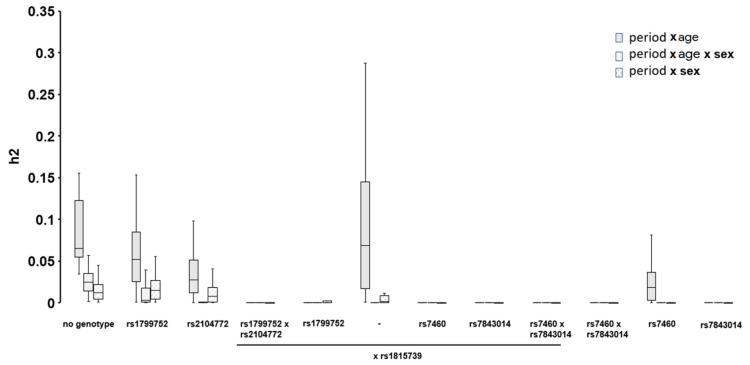
Box-whisker plots of the calculated mean effect sizes (h2) of the factors age, sex, and year and their respective interactions with genotypes, on the changes over the two periods of the skiing season as assessed by ANOVA.

***Biological influences on periodic differences in performance*:** The variability of Pmax ext 360° s^−1^, slope P × VO2-1, slope P × RER-1, and AUC lactate × P-1 was most frequently affected by either of the investigated factors of period × genotype × age × sex (Figure 5). The factor “age” exerted the strongest influence on the effect sizes of variability in changes in functional parameters over the seasonal periods, followed by the influence of the studied genotypes and sex. Figure S2 illustrates the observed effects of age on the changes in selected parameters over a seasonal period. The effect sizes of the “chronological” influence on periodic differences were above 0.1 for the following parameters: max P ext 360° s^−1^, max P ext 60° s^−1^, max HR, AUC lactate × P-1, slope P × VO2-1, and slope P × RER-1. At the post hoc level, these effects resolved in differences in the changes of assessed slopes over the preseason preparation period for the older skiers relative to the youngest skiers and maximal extension power at low angular velocity (maxP ext 60° s^−1^).

**Figure 6:**
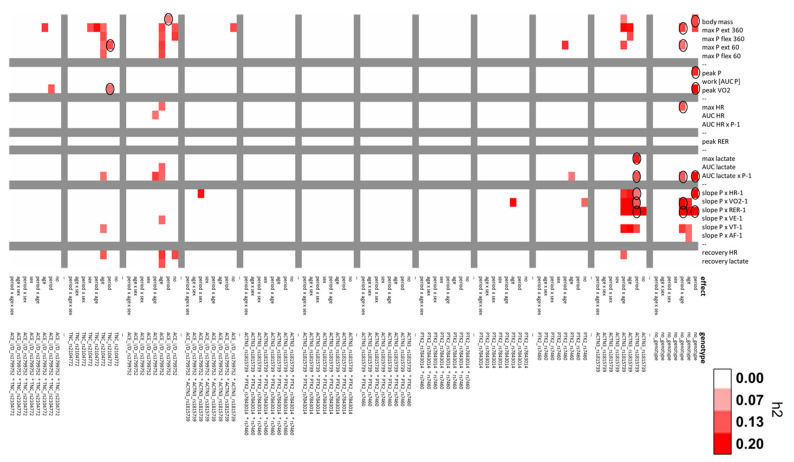
Heat map of observed effects. Color graphs summarizing the effect sizes (h2) of interaction effects of the period × age × sex and the five genotypes for changes over a respective seasonal period (ANOVA). A cut-off of 0.1 was applied, with values below being set to zero. The applied scale is given to the right. Circled effect sizes were considered relevant for the exploration of post-hoc differences.

**Figure 7:**
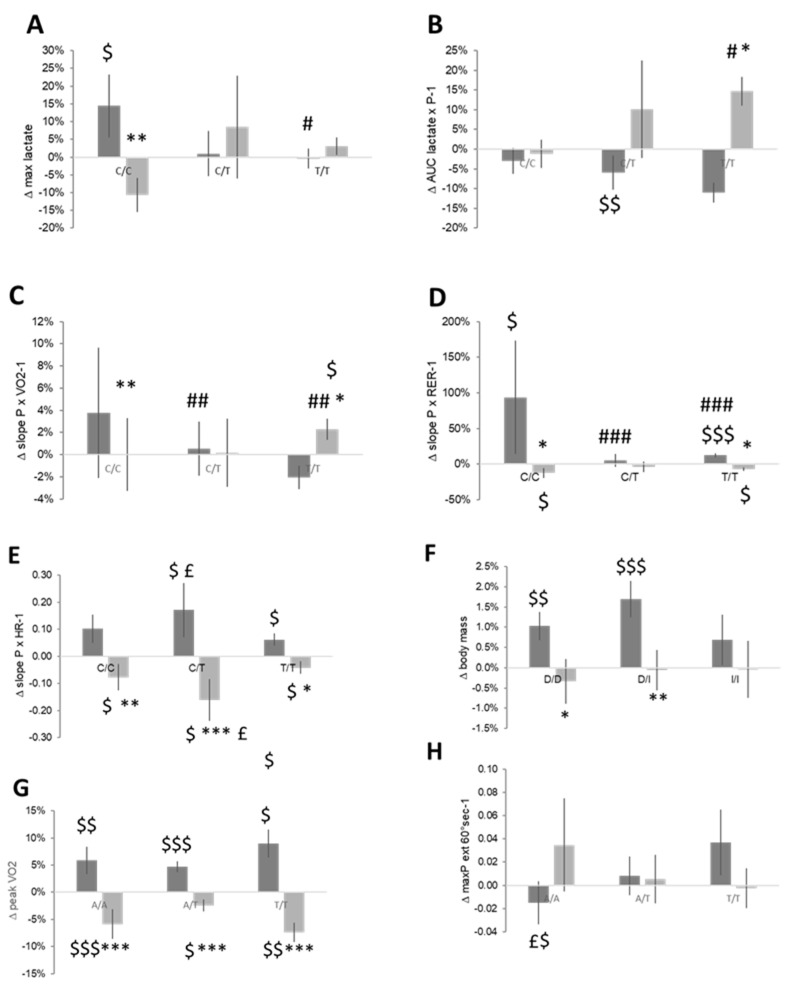
Genotype associated differences in changes over the preparation and competition periods. Bar graph of mean ± SE for the changes in performance over the preparation and competition periods of the yearly skiing season for parameters that demonstrated effects of (**A**–**E**) the ACTN3 (rs1815739), (**F**) ACE-I/D (rs1799752), and (**G**,**H**) the TNC (rs2104772) genotype. $, $$, $$$ *p* < 0.05, 0.01, 0.001 for the effect over the indicated period (sign test). *, **, ***, *p* < 0.05, 0.01, 0.001 vs. the preparation period for the same genotype. #, ##, ###, *p* < 0.05, 0.01, 0.001 vs. the CC genotype for the same period. £, *p* < 0.05 vs. TT for the same period. ANOVA with a post hoc test of least significance.

***Genotype influences on periodic alterations in performance*:** Compounded effects of the genotype on periodic variability of functional parameters were largely explained by associations with the polymorphisms for three genes, ACTN3, ACE-ID, and TNC. Eight of the resulting effect sizes for these single genetic associations with periodic changes in performance variables reached an effect size beyond 0.1 (Figure 6). These effects are presented for the following parameters that are discussed in the following section (Figure 7). Variability of periodic differences in “max lactate”, “AUC lactate × P-1”, “slope P × HR-1”, “slope P × VO2-1”, “slope P × RER-1”, and “slope P × VT-1” (not shown) were associated with rs1815739 in ACTN3, for which post hoc differences could be identified. Variability in periodic differences in body mass was associated with the ACE-I/D gene polymorphism rs1799752. Variability in periodic differences in body mass was associated with the ACE-I/D gene polymorphism rs1799752, for which post hoc differences could be resolved. Variability in periodic differences in peak VO2 and maxP ext 60 °C was associated with the TNC gene polymorphism rs2104772.

Periodic changes in muscle-related parameters, such as anaerobic strength for low and high angular velocities of extension and flexion and blood lactate concentration, were associated with rs1799752 and rs2104772, but only when the interaction with age was considered.

**Figure 8:**
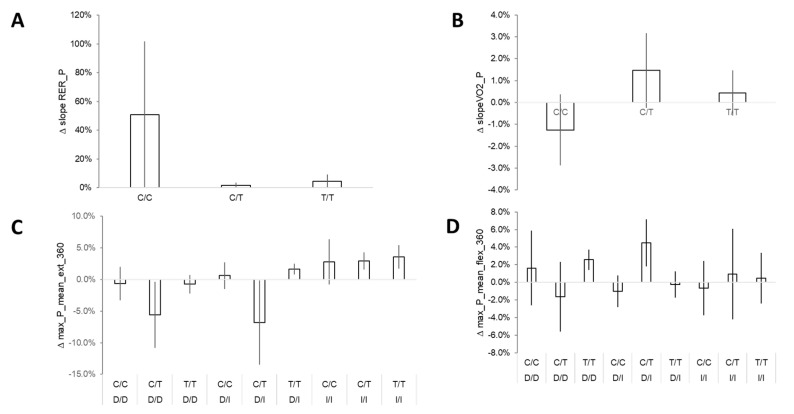
Genotype-associated differences for changes over any seasonal period. Bar graph of mean ± SE for the changes in performance over half-yearly periods of a skiing season (preparation and competition periods combined) for parameters that demonstrated effects of rs1815739 on the “slope P × RER-1” (**A**), effects of rs7460 on the “slope P × VO2-1” (**B**), and an interaction between gene polymorphisms rs1799752 × rs1815739 for “max P ext 360” and “max P flex 360” (**C**,**D**).

***Genotype-associated post hoc differences in periodic alterations in performance*:** Variability in periodic changes of four parameters was affected by genotype, irrespective of the seasonal period. The answer pattern (“reactivity”) concerned the “slope P × RER-1” for rs1815739, “slope P × VO2-1” for rs7460, and “max P ext 360” and “max P flex 360” for the interaction between gene polymorphisms rs1799752 × rs1815739 and resolved in a distinct post hoc effect (Figure 8). For rs1815739-CC genotypes, there was a highly variable periodic response in the “slope P × RER-1”. For rs7460, reduced values for the “slope P × VO2-1” after a seasonal period were observed in TT genotypes when the corresponding values were unaltered or increased in A-allele carriers. The interaction between rs1799752 × rs1815739 increased values for periodic changes in maxP extension in homozygous ACE I-allele carriers, with less favorable changes in homozygous noncarriers of the I-allele (i.e., DD genotypes) for all haplotype combinations of the ACTN3 gene polymorphism. The heterozygous haplotype ACE-ID-ACTN3-CT demonstrated the nominally largest decrease (Figure 8). Values for maxP flexion were differently associated with the rs1799752 × rs1815739 haplotype, as periodic changes were of lesser magnitude in ACE-II genotypes and most favorable in certain haplotypes, with the highest increases in the heterozygous haplotype ACE-ID-ACTN3-CT.

## 4. Discussion

The aim of this study was to elucidate the association of five polymorphisms for muscle-relevant genes with alterations in muscle performance and cardiac/muscle metabolism over the preparation and competition period of a skiing season and to assess the influence of chronological age and sex. Especially suggesting that performance alterations during the preseason and competition season periods of an entire skiing year would be affected by different gene polymorphisms. To the best of our knowledge, this is the first investigation in this field, and the results emphasize that an association exists between age, sex, and genes and the variability in periodic seasonal changes (see Supplemental File S1).

Periodic changes in maximal performance (borderline significant) (Figure 1) emphasize the deterioration of anaerobic capacity for power production over the competition period, which became more significant at low angular velocities and flexion and recovered over the preseason preparation period. Conversely, peak power (*p* = 0.07) and peak heart rate (*p* = 0.02, −0.4%) during cardiopulmonary exercise were not sensibly affected during the preseason preparation period, although maximal oxygen uptake improved and deteriorated, respectively, over the preparation and competition periods (compare Figure 1 vs. Figure 2). Interestingly, periodic changes in the slopes of produced power versus heart rate, RER, and VO2 during exhaustive exercise emphasized the worsening of metabolic capacity over the competition season period (Figure 2 and Figure 3). Alterations in these parameters are of distinct relevance for performance as they reflect a higher reliance on power production in aerobic metabolism. In the given situation of a reduced peak VO2 (over the competition period), this would be expected to produce elevations in the accumulation of blood lactate at a given high intensity of exercise. Evidence for such an adaptation is indeed identified based on measures of lactate being produced per work, i.e., AUC lactate per power (Figure 4). Collectively, the findings emphasize that the capacity of the studied elite skiers to fuel muscle work based on aerobic metabolism is not maintained over the competition period of a yearly skiing season. This also culminated in antidromic periodic changes in the work that was produced during the standardized exercise test after the competition period compared to the preparation period (Figure 1). Hence, overall, only metabolic aspects of muscle metabolism essentially improved over a skiing season.

The antidromic alterations of periodic changes in peak VO2 and maximal power during extension and flexion may explain why absolute values for these parameters were overall not improved after the four years of investigations. Interestingly, only peak RER was affected in the elite skiers after the four years of investigation with specific training and competition. This emphasizes the important influence of skiing exercise on the whole body’s metabolism. For which one can only speculate on the underlying mechanism.

Essentially, observations on seasonal subperiod-dependent alterations of power-related lactate and power-rated value slopes for metabolic parameters in elite skiers are in line with the reportedly improved O2 cost and O2 deficit during the preseason preparation of elite cross-country skiers [20]. Intriguingly, the slopes of power per ventilation and tidal volume (VE and VT, Figure 3) did not demonstrate real influences from the seasonal period. Maximal oxygen uptake is set by the serial resistances that set the oxygen transport from the lung via the cardiovasculature to skeletal muscle [38]. Observations indicate that deconditioning of aerobic metabolic capacity downstream of the pulmonary system is involved in observable changes in peak VO2 and slopes of power. The data on affected blood lactate concentration, for which skeletal muscle is considered to provide the largest portion, reflects limitations in mitochondrial oxidation of glucose [39], indicating that peripheral muscle tissue’s aerobic capacity represents an increasingly important limitation for exhaustive work after the competition season period. This may also reflect altered perfusion and oxygen extraction of muscle tissue, especially under eccentric types of contraction, which have high relevance in skiers and would essentially modify metabolic efficiencies [13,37,38]. This is particularly interesting as eccentric contractions can enhance the oxygen deficit of recruited leg muscles and since the volume content of mitochondria is known to deteriorate after endurance-type training under eccentric load [40,41,42]. The underlying mechanism is not fully elucidated but may involve a reduced haemoglobin content due to the underperfusion of muscle that is strained with intense eccentric contraction, and this may lead to possible mechanical damage that necessitates an adaptive cycle to repair and re-establish damaged cell structures, including mitochondria [40,43,44].

The intention of the preseason preparation period for the main competition season period is to increase aerobic capacity since this is understood to improve recovery from exercise, as this is identifiable in a more rapid fall in cardiac output or heart rate [45]. Indeed, we noted measurable increases in heart rate during recovery from exercise after the competition season period (Figure S1). This would be expected to reflect reduced metabolic reserves in the athletes after the competition season period, which may limit the performance that can be sustained during a physical challenge that follows shortly after the first exhaustive exercise.

Influences of age, as identified here for periodic changes (Figure 5), have been noted before regarding the development of muscle metabolism-related sports performance, especially variables of aerobic metabolism and strength [46,47]. Absent evidence for a difference in age between the years of the studied window of investigation (*p* = 0.819, Figure S3) advocates against a major contribution of a drift due to changes in cohort composition or yearly differences in the training stimulus but rather a role of biological age to the observed physiological changes during seasonal periods. In this respect, the larger increases for the slope P × VO2-1 and slope P × RER-1 over the preseason preparation period in the athletes aged between 15 and 18 years (Figure S2) meet the expectation of an elevated contribution of respiratory processes to power production during (sub)maximal exercise when pubertal athletes grow older [48].

Likewise, the larger decline in maximal power at high angular velocity of extension (i.e., maxP ext 360 × s^−1^) over the competition season period of the athletes in the age category between 25–30 years of age, compared to those of 21–22 years of age, is in line with the notion that muscle isometric and dynamic strength typically increased up to the third decade, with evidence for a fibre composition-related onset of losses in knee extensor muscle cross sectional area (i.e., type II fibre atrophy) at 25 years of age [49,50]. In this respect, the larger improvement in maximal power at low angular velocity (maxP ext 60° s^−1^) in the 25–30 year age group compared to the 15–18 year age group is of interest, as this is expected to be related to slow-type muscle characteristics, which are expected to increase relatively with increasing age. In the specific situation of systematic training, however, a portion of the age-related variability of parameters that characterize aerobic metabolism can possibly be attributed to an increased volume and intensity of specific training for the youngest elite skiers as they enter a (semi) professional occupation in sport when they are admitted into the skiing squad. Conversely, it may be speculated that the training loads may be lower for senior athletes. Overall, the observations point to a considerable influence of age-related human biology on improvements in aerobic performance in junior elite athletes (15–18 years of age) and a decline in anaerobic performance in senior studied athletes (25–30 years of age).

With respect to the influence of age on periodic alterations in muscle performance, the largely age-dependent associations between periodic changes in anaerobic strength for low and high angular velocities of extension and flexion with gene polymorphisms rs1799752 and rs2104772 are intriguing (Figure 6). The investigated gene polymorphism indeed affects the expression and amino acid sequence, respectively, of the encoded proteins ACE and TNC (reviewed in [10,31,34]), both of which are implicated in the age-dependent loss of the fast fibre population, which contributes to the dynamometrically recorded maximal values in leg strength [51]. These relationships point to a relevant contribution of ACE and Tenascin-C-modulated myogenic processes to the variable outcome of the physical conditioning of elite skiers during the preparation period on anaerobic strength.

Sex was also associated with variability in periodic differences for certain physiological parameters (only one, i.e., the “slope of P × HR-1”, demonstrated an association of periodic changes with sex in interaction with the combination of rs1799752 × rs1815739 genotypes, which passed the threshold of 0.1 for the effect size, Figure 6); genotype interactions were identified to demonstrate more notable effect sizes.

Regarding genetic influences, it is striking that the rs1815739-associated variance of periodic changes exclusively concerned parameters that reflect relationships between produced muscle power and metabolism during exhaustive exercise, i.e., max lactate, AUC lactate × P-1, slope P × HR-1, slope P × VO2-1, and slope P × RER-1, but not muscle power per se. All these parameters reflect the contribution of the addressed metabolic process to the generation of mechanical output [33]. We observed a pattern where the magnitude of the difference in antidromic periodic changes in maximal blood lactate, power-related blood lactate (AUC lactate × P-1), and the slope P × RER-1 over the preseason preparation and competition season periods was proportional to the copy number of the ACTN3 T-allele (Figure 7A,B,D). On closer inspection, the observed “reciprocity” of the ACTN3-C/T-genotype associated funnel effect in the differences in periodic changes between preseason preparation and competition season periods for maximal blood lactate and power-related blood lactate was explained by an influence of the ACTN3 genotype on the work being performed during CPX testing, which did not demonstrate a sufficiently large effect size to pass the applied statistical threshold, i.e., h2 = 0.007). The previously mentioned parameters are reflective of the capacity to sustain glycolytic strain during exhaustive exercise (reviewed in Schmutz et al., 2010 [39]). The findings corroborate the view that the gene polymorphism rs1815739 is related to the metabolic efficiency of skeletal muscle [52] via influences on the differentiation of muscle fibre type through alterations in the amino acid sequence of the encoded proteins [53]. The specifically observed differences argue for a largely more fluctuating aerobic phenotype in ACTN3-CC genotypes over a skiing season, i.e., higher degrees of deterioration over the competition period, which is consistent with the view that this genotype favors the development of metabolically less economic type II muscle fibres with reduced aerobic capacity [30]. The collaterally observed conversely higher degree of difference in periodic changes for work-related blood lactate accumulation (AUC lactate × P-1, Figure 7B) indicates that the ACTN3 genotype-associated changes in aerobic must be seen in relation to the deterioration of the work capacity over the competition season period. In this respect, the observation that variability in periodic changes in maximal lactate only manifested when interactions with rs1815739 were considered (thereby increasing effect size 7-fold) is suggestive that the ACTN3 genotype may exert an important contribution to seasonal changes in blood lactate. The latter parameter is often used in combination with heart rate and oxygen uptake to monitor exercise intensity and dose the metabolic effect of physical conditioning in elite skiers [16,54,55], which, if used in combination, allows us to explain individual differences in the response to a training paradigm [56]. In regard to the latter technical aspect, it is striking that peak values for maximal heart rate, similar to maximal lactate, did not demonstrate strong intraseasonal variation (i.e., periodic changes), except when the factors of age and genotype were considered (Figure 2 and Figure 6). In contrast, considerable periodic changes resolved the contribution of metabolic parameters to power development. This emphasizes that the latter factors that are indicative of “metabolic efficiencies” and oxygen deficits [20] may be better suited to resolve adaptations of skeletal and cardiac muscle metabolism with periods of physical conditioning.

Noting specific decreases in the maximal power of slow leg extensions (Pmax ext 60° s^−1^) over the preseason preparation period in rs2104772-AA genotypes, homozygous rs2104772-T-allele carriers demonstrated a different trend, i.e., improvement (Figure 7H). This observation highlights important rs2104772-related differences in the strength response to the provided “training stimuli”. Additionally, noting the association of the gene polymorphism rs2104772 with variability in periodic changes in aerobic capacity (peak VO2) in elite skiers (Figure 7G), this association could not be localized post hoc. We and others have previously identified that rs2104772 is associated with differences in capillary length density and muscle fibre cross-sectional area in endurance and strength-type athletes [10,57].

Likewise, an effect of the gene polymorphism rs1799752 existed regarding the periodic changes in leg extension and flexion strength at low and high angular velocities (i.e., “max P ext 360” and “max P flex 360”), which were independent of the respective periods. This finding reproduces the reported influences of rs1799752 gene polymorphisms on the fibre type distribution, volume densities of myofibrils, and mean cross-sectional area of vastus lateralis muscle fibres [10,34]. D-allele carriers of the ACE gene also demonstrated increased changes in body mass over the preseason preparation period (Figure 7F). Associations between ACE-I/D and changes in fat and lean mass during rigorous training of young male subjects have been reported before and appear to be related to differences in metabolic efficiency [28]. This is possibly also related to differences in muscle cross-sectional area, which was conversely higher in untrained and trained homozygous ACE-I allele carriers [34]. We do not know how the increased body mass explains itself, i.e., lean or fat mass, but it is tempting to speculate that it is either or both. The rs1799752 genotype alone was also associated with periodic post-exercise changes in the recovery of heart rate (Figure 6), which is greatly in line with the potent influence of ACE-modulated cardiac performance [28].

The investigation did not demonstrate major associations between the studied gene polymorphisms in PTK2 and variability in periodic changes in physiological parameters that are associated with aerobic or anaerobic strength. As recently reported, elite skiers demonstrate PTK2 gene polymorphism rs7843014-associated variability in leg muscle strength and pulmonary ventilation [26]. These findings emphasize that specifically PTK2-associated endpoints, such as a specific muscle force or stiffness [24], should be assessed in future studies [57,58,59]. Importantly, while athletes progressively markedly improved only aspects of whole-body respiration over the investigated four skiing seasons (Table 1), variability in changes in certain performance indicators over the half-yearly periods of a skiing season (preparation or competition periods combined) demonstrated genetic effects (Figure 8). Foremost, this concerned an influence of the interaction between gene polymorphisms rs1799752 × rs1815739 on the variability of periodic changes of the maximal performance in extension and flexion at high velocity, i.e., “max P ext 360” and “max P flex 360”. Therefore, the reactivity of Pmax in extension and flexion was differentially affected in homozygous T-allele carriers and noncarriers of the gene polymorphism rs1815739, whereby this was conversely modulated in the heterozygous rs1799752-ID- × rs1815739-CT haplotypes. Interpreting the further highly variable periodic response in the “slope P × RER-1” between rs1815739 genotypes as another example of the discussed role of ACTN3 in affecting the metabolic efficiency of skeletal muscle [51]. The further identified reduced values for the periodic changes of the “slope P × VO2-1” in TT genotypes compared to A-allele carriers of rs7460 may be an example of the genetic influence on (muscle) tissue stiffness [54], which may affect aerobic metabolic efficiency. Collectively, our data extend the knowledge on adaptations in strength with strength training in association with the ACTN3 genotype [56] by showing that the interaction with the ACE-ID genotype needs to be considered a modulatory factor (Figure 8).

Overall, the genetic influences on performance adaptations identified here are in line with the reported implications of the studied polymorphisms with variability of exercise-regulated muscle plasticity. This especially holds true for the influences of gene polymorphism in the ACE, ACTN3, and TNC genes on the expression of the encoded proteins. The observed post hoc differences in periodic changes in a number of performance parameters in association with gene polymorphisms rs1799752, rs1815739, and rs2104772 are in line with the expected influences (i.e., gain or loss of function) from the affected protein product encoded by the polymorphic gene. For instance, the rs2104772-associated reactivity in Pmax during slow leg extension and peak VO2 is consistent with the involvement of TNC in the regulation of fast muscle fibre type growth and angiogenesis of muscle tissue through exercise-intensity modulated TNC expression in skeletal muscle [56,57,58,59,60,61]. Equally, the studied PTK2 gene polymorphisms are in line with potential influences of the encoded protein on the metabolic efficiency of exercising skeletal muscle through possible influences on biomechanical aspects of tissue function.

The investigation has a number of limitations that need to be considered when interpreting the data. First, repetitively assessing subjects from different disciplines through consecutive skiing seasons to increase statistical power, but knowing little about the exact nature of the deployed training regimes—except the systematic contribution of strength-type exercise, including a high proportion of slow isometric contractions under a high load type in the preseason preparation period—cannot explain the functional gains and losses in terms of the specific training being imposed. Last, values from laboratory tests are only one factor to possibly explain variability in skiing performance (and are limited by the number of subjects from the squad of elite skiing athletes willing to enter the investigations) and only incompletely predict success in competition [16]. Additionally, note that physiological parameters of performance are, although relevant for the capacity and safety factors to apply technical skills demanding manoeuvers on skis, not alone responsible for the performance in ski races. Therefore, not attempting to evaluate the degree to which seasonal variation in physical aspects is monitored in a standardized laboratory situation may translate into skiing-specific motor-control skills and performance.

In regard to methodological aspects, noting the unusual/abnormal, i.e., nonnormal, behavior of mean vs. median values for certain parameters, such as the periodic change for “AUC heart rate” and the “slope P × RER-1” over the preparation period, motivates the (general) use of the nonparametric sign-test to identify changes post vs. pre-seasonal period. Therefore, periodic effects were resolved, for which median and mean values did not appear to differ largely before and after a seasonal period.

To summarize, the observation can have bearing on the sophistication and design of personalized approaches to maximize the conditioning of physical performance in elite skiers (or athletes in general) and the mitigation of losses in anaerobic strength and skeletal and cardiac metabolic capacity in periods when conditioning stimuli are reduced due to constraints of competition. Routine testing of “maximal values for certain functional variables” appeared to be of lesser relevance to resolving fluctuations in performance during seasonal periods than considering the athletes’ age and sex and knowing the identification of assessed genotypes. Genetic information appears to constitute a relevant complement of information to assist coaches by providing elite skiers with better individualized advice on how to maintain and improve performance during seasonal periods.

## 5. Perspective

A pattern of improvement during the preseason preparation period in the summer could be identified. The improved capacity may allow athletes to withstand the high loads during the competition period in the winter and prevent performance drops or injuries. In this context, physical fitness and fatigue-related factors, for instance, are considered key factors for injuries in competitive alpine skiing. Future studies should therefore investigate more specifically the gene variant-induced seasonal variations in muscle strength and aerobic capacity as a potential cause of injury.

The detected decline during the competition season period in the winter months was age, genotype, and sex dependent. Changes in aerobic performance were larger (6%) than those in anaerobic extension and flexion leg strength (3%). Therefore, it is suggested that increasing aerobic performance might be beneficial. Depending on the specific genotype, training stimuli could be designed in the future when the interaction of genotypes and performance characteristics is sufficiently understood. Generally, parameters of aerobic performance might be improved in the preseason preparation period to build up reserves and better maintain a higher level during the following competition season. Thereby, HIIT training might be especially suitable, allowing these high-level athletes to improve in a short time. As strength and endurance training in high-level athletes interfere, block periodization is recommended to have the maximum effects of training stimuli in the shortest time. A three-week block of three 4 × 4 min HIIT training sessions (65–85% VO2max) might be an adequate measure.

## Figures and Tables

**Table 1:** Physiological characteristics of elite skiers. Mean + SE of physiological parameters as assessed before the preparation phase in the first and last years of study, i.e., 2015 and 2018, during isometric strength testing and cardiopulmonary exercise for the thirty-four study participants for whom data over consecutive seasonal phases were available. *p*-values were calculated for the difference between the two timepoints (ANOVA). Underlined values were deemed significant.

	2015-Pre Comp					2018-Pre Comp			
	Mean	SD	Min	Max	*p*-Value	Mean	SD	Min	Max
**Biometry**									
age [years]	20.4	2.9	16.2	26.9	**0.021**	22.3	2.8	18.6	29.9
height [cm]	174.1	8.6	155	200	0.879	174.5	9.9	156	201
body mass [kg]	71.8	10.5	56.7	102.5	0.956	71.6	13.4	56.8	106.1
**Anaerobic performance**									
Pmax extension 60° s^−1^ [W]	209.8	50.2	156.4	376.5	0.49	222.7	55.9	134.6	343.1
Pmax extension 360° s^−1^ [W]	677.4	196.4	435.4	1230.5	0.541	721.3	209.3	490.8	1177.8
Pmax flexion 60° s^−1^ [W]	123.2	37.8	81.5	239.8	0.825	126.3	40.4	85.5	205
Pmax flexion 360° s^−1^ [W]	461.8	170.4	286.5	939	0.573	495.3	159.5	311.5	890.9
**Aerobic performance**									
VO2max [L min^−1^]	3.6	0.8	2.4	5.3	0.759	3.6	0.8	2.4	5.1
peak P [W]	316.7	58.6	250	430	0.585	306.8	69.6	190	434
peak HR [bpm]	194.4	10.1	178	215.1	0.647	193.1	8.2	180.3	215
peak RER	1.64	0.19	1.2	2.05	**<0.001**	1.41	0.08	1.3	1.58
peak lactate [mM]	11.9	2.4	8.2	16	0.284	11.1	2.8	4.8	15.9

## Data Availability

Data is available on qualified request.

## Supplementary Materials

The following Supplemental tables and the supplemental figures can be downloaded https://www.mdpi.com/article/10.3390/genes14061165/s1; Figure S1. Box Whisker plots of the changes during recovery from exhaustive exercise over the preparatory and competition periods of the yearly skiing season. $$$, *p* < 0.001, for the effect of the seasonal period (sign test). * *p* < 0.05 vs. the preparation period. ANOVA with post-hoc test of least significance. Figure S2: Influence of age on alterations over the preparation and competition period of a skiing season. Box Whisker plots of the changes over the preparation and competition period in different age categories for parameters with age × period interactions. *, **, *** *p* < 0.05, *p* < 0.01 and <0.001 vs. the preparation period for same age. $, *p* < 0.05 vs. age 15–18 years over the same period. #, *p* < 0.05 vs. 25–30 years for the same period. Figure S3: Athlete age over the studied years of training. Box-Whisker plots of subject age in the preparation and competition period of the consecutively studied training years. Figure S4: Representative examples of the original output from difference melt plots used to identify genotypes for the selected polymorphim rs1815739 (A), rs7843014 (B), rs7460 (C), and rs2104772 (D) based on high resolution melt analysis for the PCR products. Supplemental File S1: Research assumptions; Supplemental Table S1: Reaction conditions for the detection of selected gene polymorphisms with PCR. Reactions were carried out with 10 ng genomic DNA and specific combinations of 0.2 μM primers and 2.5 mmol MgCl2 using KAPA HRM FAST Master Mix (KAPA BIOSYSTEMS) on a real-time PCR system (EcoTM, Illumina, San Diego, USA).

## Abbreviations

ACE_I/D	insertion/deletion gene polymorphism of angiotensin converting enzyme
ACTN3	actinin-3
AF	breathing frequency during CPX
AUC	integral of values as calculated by the area under the curve during CPX
AUC HR	AUC of heart rate during CPX
AUC HR × P-1	AUC of heart rate during CPX per conducted power
AUC lactate	AUC of blood lactate concentration during CPX
AUC lactate × P-1	AUC of blood lactate concentration during CPX per conducted power
AUC P	work performed during CPX calculated by the time integral of power
CPX	cardiopulmonary exercise
heart rate	heart rate
max HR	maximal heart rate
max lactate	maximal values of blood lactate concentration
max P ext 360	maximal power during leg extension at an angular velocity of 360° s^−1^
max P flex 360	maximal power during leg flexion at an angular velocity of 360° s^−1^
max P ext 60	maximal power during leg extension at an angular velocity of 60° s^−1^
max P flex 60	maximal power during leg flexion at an angular velocity of 60° s^−1^
peak P	peak power output during CPX
peak RER	peak respiration exchange ratio
peak VO2	peak oxygen uptake during exhaustive cardiopulmonary exercise
PTK2	protein tyrosine kinase 2
RER	respiration exchange rate
slope P × AF-1	slope of correlation between power output and AF during CPX
slope P × HR-1	slope of correlation between power output and HR during CPX
slope P × RER-1	slope of correlation between power output and RER during CPX
slope P × VE-1	slope of correlation between power output and VE during CPX
slope P × VO2-1	slope of correlation between power output and VO2 during CPX
slope P × VT-1	slope of correlation between power output and VT during CPX
TNC	tenascin-C
VE	minute ventilation [L min^−1^]
VT	tidal volume [L]
VO2	oxygen uptake
recovery HR	delta between 2 min after and abortion of CPX for HR
recovery lactate	delta between 2 min after and abortion of CPX for blood lactate
