# Supplementary material for: Association of Gene Variants with Seasonal Variation in Muscle Strength and Aerobic Capacity in Elite Skiers
Source: Genes (Basel). 2023 May 26;14(6):1165. doi: 10.3390/genes14061165 (PMC10298717; doi:10.3390/genes14061165)
Supplement: Supplementary file 1 [file genes-14-01165-s001.zip › genes-2382312-supplementary/genes-2382312-Supplemental File S1s.pdf]

Supplemental File S1: *Research assumptions*

-Phasic changes in parameters that reflect metabolic efficiency would be affected by the interaction between ACTN3 and ACE genotype, though influences on lactate accumulation, heart rate and VO2. Thereby the power-related accumulation of lactate during exhaustive exercise would be expected to be less in ACTN3 TT genotypes than CC genotypes in possible interaction with the ACE I/D-gene polymorphism, especially since I-allele carriers show increased mitochondrial biogenesis with training and better capacity for perfusion (1),(2),(3).

-Muscle performance would be differently affected between ACTN3-CC genotypes than TT genotypes owing to the association with fiber type composition (4).

-Gene polymorphism of TNC would be associated with different phasic changes in maximal performance and oxygen uptake due to the influence of the encoded protein in muscle growth and repair and angiogenesis (5),(6),(7).

-PTK2 gene polymorphism may affect muscle performance owing to the influence on muscle stiffness (8) and this may also involve auxiliary respiratory muscle.
