# Supplementary material for: Association of Gene Variants with Seasonal Variation in Muscle Strength and Aerobic Capacity in Elite Skiers
Source: Genes (Basel). 2023 May 26;14(6):1165. doi: 10.3390/genes14061165 (PMC10298717; doi:10.3390/genes14061165)

Figure S1. Box Whisker plots of the changes during recovery from exhaustive exercise over the preparatory and competition periods of the yearly skiing season. $$$, p<0.001, for the effect of the seasonal period (sign test). * p<0.05 vs the preparation period. ANOVA with post-hoc test of least significance.

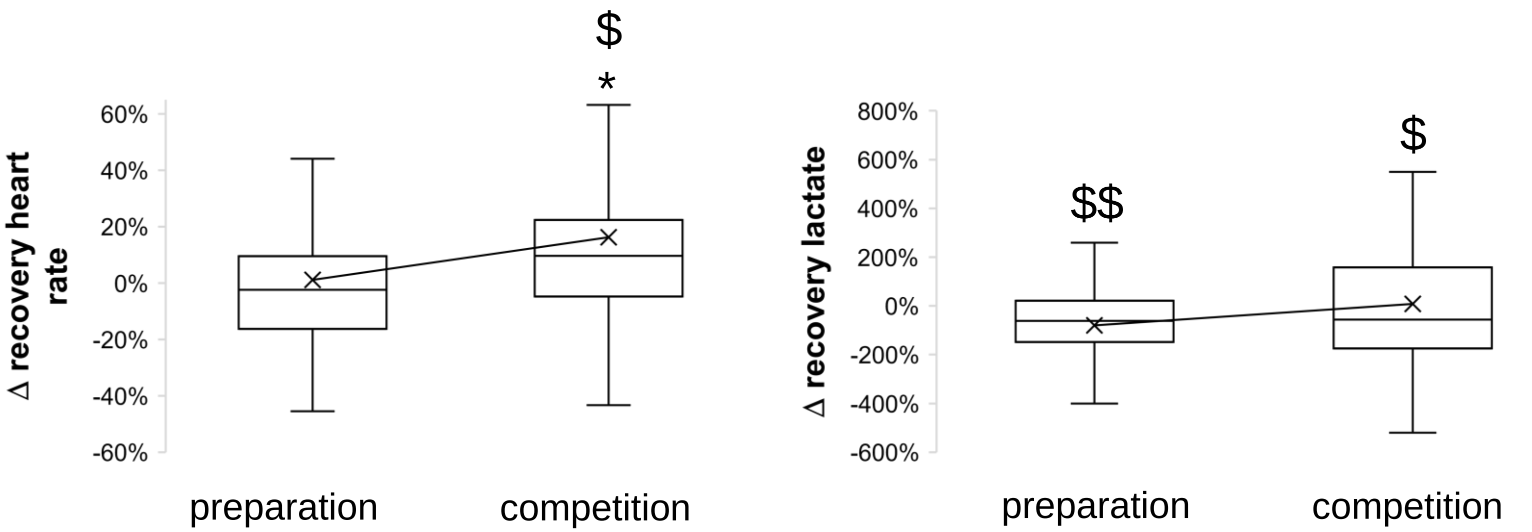

Figure S2: *Influence of age on alterations over the preparation and competition period of a skiing season.* Box Whisker plots of the changes over the preparation ( ) and competition period ( ) in different age categories for parameters with age x period interactions. *, **, *** p<0.05, p<0.01 and <0.001 vs the preparation period for same age. $, p<0.05 vs age 15-18 years over the same period. #, p<0.05 vs 25-30 years for the same period.

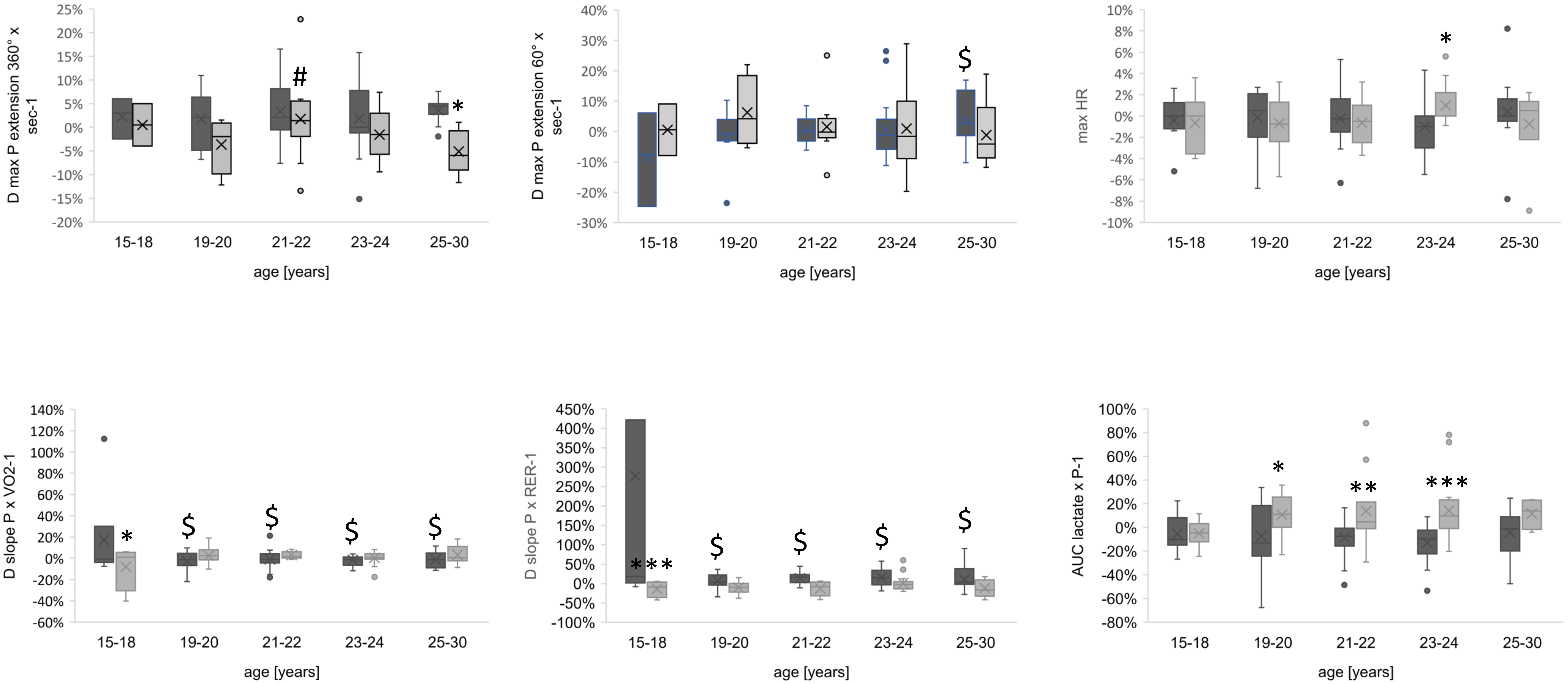

Figure S3: *Athlete age over the studied years of training.* Box-Whisker plots of subject age in the preparation ( ) and competition period ( ) of the consecutively studied training years.

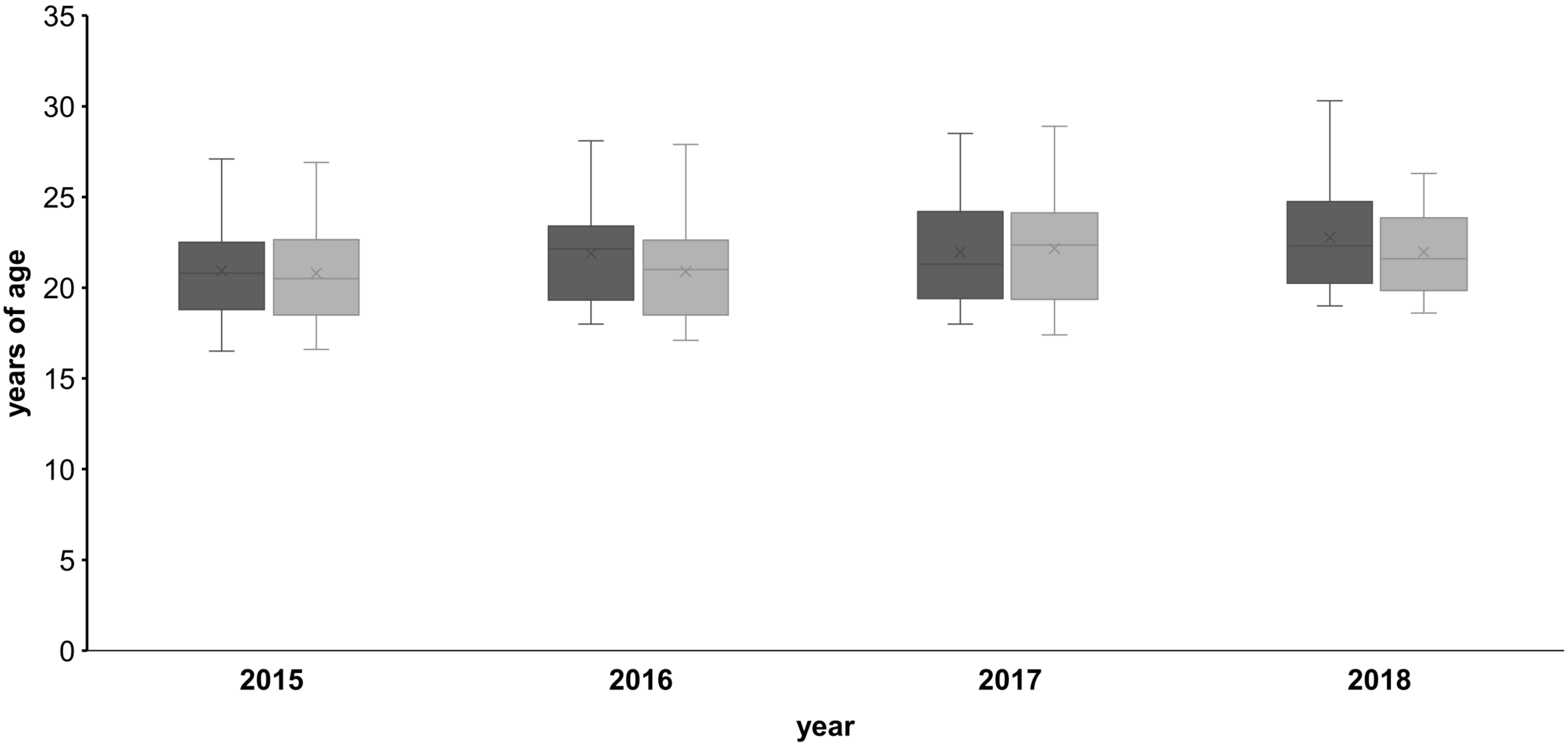

Figure S4: Representative examples of the original output from difference melt plots used to identify genotypes for the selected polymorphim rs1815739 (A), rs7843014 (B), rs7460 (C), and rs2104772 (D) based on high resolution melt analysis for the PCR products.

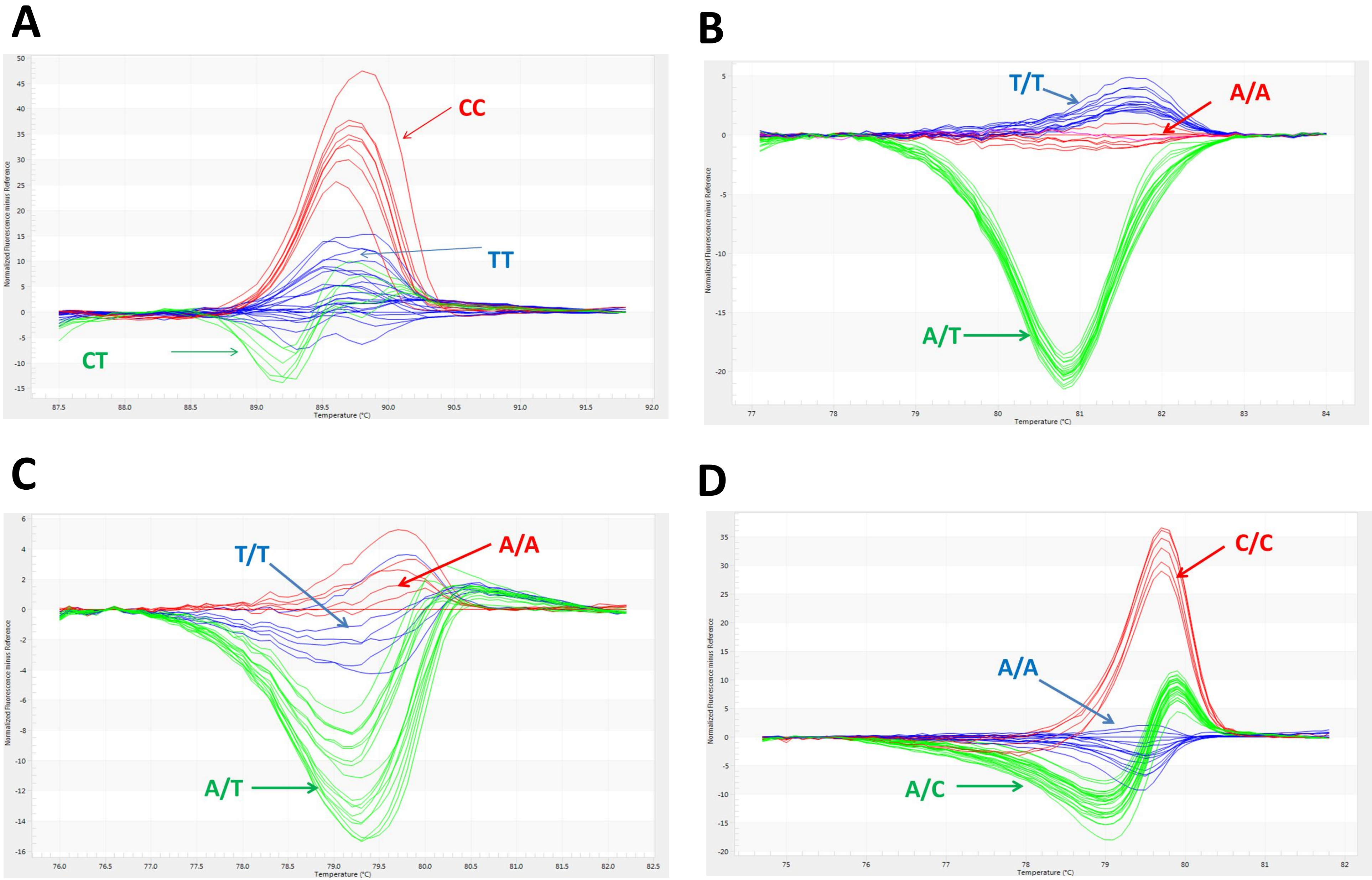

Supplement: Supplementary file 1 [file genes-14-01165-s001.zip › genes-2382312-supplementary/genes-2382312-supplementary Figures.pdf]
