# Supplementary material for: Association of Gene Variants with Seasonal Variation in Muscle Strength and Aerobic Capacity in Elite Skiers
Source: Genes (Basel). 2023 May 26;14(6):1165. doi: 10.3390/genes14061165 (PMC10298717; doi:10.3390/genes14061165)
Supplement: Supplementary file 1 [file genes-14-01165-s001.zip › genes-2382312-supplementary/genes-2382312-supplementary Table S1.pdf]

Table S1. Reaction conditions for the detection of gene polymorphims with PCR. Reactions were carried out with 10 ng genomic DNA and specific combinations of 0.2 μM primers and 2.5 mmol MgCl2 using KAPA HRM FAST Master Mix (KAPA BIOSYSTEMS) on a real-time PCR system (EcoTM, Illumina, San Diego, USA).

Gene	*Polymorphism*	*Forward primer*	*reverse primer*	*thermal protocol*
ACE	rs1799752 - I allele	5′-tgggattacaggcgtgatacag-3′	5′ atttcagagctggaataaaatt-3′	3 min at 95°C; 35 x (5 sec at 95°C, 30 sec at 55°C), melting cycle (95°C, followed by ramp from 55°C - 95°C, 0.3°C sec-1)
ACE	rs1799752 - D allele	5′-catcctttctcccatttctc-3′	5′-atttcagagctggaataaaatt-3′	3 min at 95°C; 35 x (5 sec at 95°C, 30 sec at 55°C), melting cycle (95°C, followed by ramp from 55°C - 95°C, 0.3°C sec-1)
ACTN3	rs1815739	5′-ctgtttgcctgtgtgtaagtggggggg-3	5′-tgtcacagtatgcaggagggg-3′	3 min at 95°C; 35 x (5 sec at 95°C, 30 sec at 60°C), melting cycle (95°C, followed by ramp from 55°C - 95°C, 0.3°C sec-1)
PTK2	rs7460	5′-tgggtcgggaactagctgta-3′	5'-atggaaaaaggggatggtcc-3'	3 min at 95°C; 35 x (5 sec at 95°C, 30 sec at 60°C), melting cycle (95°C, followed by ramp from 55°C - 95°C, 0.3°C sec-1)
PTK2	rs7843014	5'-tgatgggacctaaacccatt-3'	5'-tttcccatcagctgcttgtt-3'	3 min at 95°C; 35 x (5 sec at 95°C, 30 sec at 60°C), melting cycle (95°C, followed by ramp from 55°C - 95°C, 0.3°C sec-1)
TNC	rs2104772	5′-caaaaagcagtctgagccac-3′	5′-ttcagtagcctctctctgagac-3′	3 min at 95°C; 35 x (5 sec at 95°C, 30 sec at 60°C), melting cycle (95°C, followed by ramp from 55°C - 95°C, 0.3°C sec-1)
